# Excess Mortality Stratified by Age and Sex for Croatia and Croatian Counties during the 2020–2021 COVID-19 Pandemic

**DOI:** 10.3390/idr16020011

**Published:** 2024-02-20

**Authors:** Mara Šošić, Zvonimir Boban, Marijan Erceg, Nataša Boban

**Affiliations:** 1 Department of Clinical Epidemiology, University Hospital of Split, 21000 Split, Croatia; mara.kavelj@kbsplit.hr; 2 Department of Medical Physics and Biophysics, University of Split School of Medicine, 21000 Split, Croatia; zvonimir.boban@mefst.hr; 3 Division for Epidemiology and Prevention of Noncommunicable Chronic Diseases, Croatian Institute of Public Health, 10000 Zagreb, Croatia; 4 Department of Public Health, University of Split School of Medicine, 21000 Split, Croatia

**Keywords:** COVID-19, excess mortality, excess mortality percentage change, excess mortality rate, Croatia, age, sex, county, undercount, population density

## Abstract

Excess mortality is often used to estimate the effect of a certain crisis on the population. It is defined as the number of deaths during a crisis exceeding the expected number based on historical trends. Here, we calculated excess mortality due to the COVID-19 pandemic for Croatia in the 2020–2021 period. The excess was calculated on the national and county level for different age and sex categories. In addition to the absolute number, the excess mortality was also expressed as a ratio of excess deaths to the predicted baseline and excess mortality rate. We showed that using both measures is necessary to avoid incorrect conclusions. The estimated excess mortality on the national level was 14,963, corresponding to an excess percentage of 14.3%. With respect to sex, there was a higher excess mortality rate for men compared to women. An exponential relationship was observed between age and the excess mortality rate.These trends wee representative of most counties as well, with large variations in the magnitude of the effect. However, there were also exceptions to the general rule. The reasons for these deviations were discussed in terms of between-county differences in demographic structure, population density and special events that took place during the pandemic.

## 1. Introduction

The COVID-19 pandemic, caused by the novel coronavirus SARS-CoV-2, was first identified in Wuhan, China, towards the end of 2019. It quickly escalated into a global health emergency, with the World Health Organization declaring it a pandemic in March 2020. This crisis tested public health systems worldwide, disrupted international travel and led to significant economic and social challenges.

The impact of an infectious disease is traditionally estimated based on the number of cases and the number of deaths. However, both of these metrics can be greatly influenced by the limited availability of testing and different definitions of COVID-19 deaths used by different countries [1]. For example, some countries only count PCR-confirmed COVID-19 deaths, while others, such as Croatia, include suspected COVID-19 deaths as well. Additionally, even if counted perfectly, the official number of deaths from COVID-19 only provides a measure of the direct impact of the pandemic and does not take into account the indirect effects on total country mortality. These effects might include deaths caused by the collapse of the medical system, by the psychological effects of isolation, etc.

This is why excess mortality emerged as a crucial indicator during the COVID-19 pandemic [1]. This metric shows the number of deaths exceeding reference levels calculated based on historical mortality data. Assuming the incidence of other diseases remained stable over the period of interest, excess mortality can give us an insight into the number of deaths directly or indirectly related to COVID-19.

Excess mortality can be displayed just as an absolute number of excess deaths, but more commonly, it is presented as relative excess mortality, or specific excess mortality rate. Relative excess mortality is defined as the change in mortality relative to an expected mortality value calculated based on data from some reference time frame. It is often used to express excess mortality since it can be calculated solely from the mortality data and provides information about the relative change in mortality due to an event. However, it may not be suitable in certain situations, such as those where the baseline mortality rates are extremely low, so small changes can result in very high relative excess mortality values that may be misleading. Additionally, it does not explicitly account for the population size, making it harder to compare the absolute impact of an event in different locations. Excess mortality rate addresses this issue by standardizing the absolute excess mortality using population size, but does not include information on the relative impact of those additional deaths compared to the baseline. Consequently, this study will use both metrics in order to obtain a more complete picture, capturing both the relative change in mortality provided by the excess percentage and the absolute impact of the event provided by the excess rate.

To provide a global perspective on excess mortality during the COVID-19 pandemic, excess mortality calculations for the total country populations (including Croatia) have already been performed by other studies such as the one by Karlinsky and Kobak [2] and the COVID-19 Excess Mortality Collaborators [3]. However, both of those studies were based on the World Mortality Dataset, which possibly included preliminary data subject to future revisions, potentially impacting the overall accuracy. Moreover, since exact population data are not available for each year for every country, the Karlinsky and Kobak study relied on the population size estimates for 2020 from the United Nations World Population Prospect (WPP) dataset [4]. Finally, because of their global scope, the above studies do not include calculations over different age–sex subgroups (on national and subnational levels), and these differences must be considered in order to fully understand the differences in the effects of the COVID-19 pandemic on a certain population. We also found a single study on excess mortality focusing on Croatia [5], but the excess mortality was calculated there only for the year 2020, and only at the national level, without accounting for variations across different counties.

The COVID-19 pandemic had varying impact on different segments of the population, particularly when considering factors like age and sex. Older adults were at much higher risk of severe outcomes compared to younger people [6,7], with an exponential relationship being observed between age and infection fatality rate [7]. With respect to age, there seems to be a significant male bias in mortality from COVID-19 [8,9,10], with an estimated average female/male mortality incidence ratio of 0.7 across European countries.

The goal of this retrospective observational population-based study is to calculate excess mortality in Croatia for the 2020–2021 period based on historical data from the 2016–2019 period. The excess will be calculated separately for different counties and age and sex groups. The variations in excess mortality will be discussed in terms of between-county differences in demographic structure, population density and special events that took place during the pandemic.

## 2. Materials and Methods

The data used in this study were obtained from the Croatian Bureau of Statistics and Croatian Institute of Public Health [11]. The epidemiological indicator used was excess mortality, defined as the difference between the actual and expected number of deaths in a certain time period. In addition to the cumulative absolute excess mortality, we also expressed the excess as relative excess mortality and excess mortality rate per 1000 people. The specific mortality rates per 1000 people were obtained by dividing the absolute excess mortality by the number of people belonging to a certain age and sex subcategory and multiplying by thousand. Expressed mathematically, $Relative\;excess\;moratlity=\frac{Excess\;mortality}{Expected\;mortality}$, and $Excess\;mortality\;rate\;per\;1000\;people=\frac{Excess\;mortality}{Category\;population\;size}\times 1000$. The number of people in each subcategory was obtained from the 2021 Census of Population performed by the Croatian Bureau of Statistics [12]. The population was stratified into four age (0–64, 65–74, 75–84 and 85+) and two sex (male, female) categories. 

The excess mortality was calculated for the 2020–2021 period based on the baseline predictions obtained using the mortality data from the 2016–2019 period. For estimation of the baseline, we adopted the methodology from Karlinsky and Kobak [2]. Briefly, the baseline for different counties was modeled using a linear regression model with a common slope coefficient, but a different intercept for each month, or expressed mathematically—$D_{month,\;year}=\beta \cdot year+\alpha_{month}+\epsilon$. This is the simplest model that takes into account both the between-year (through the slope coefficient) and seasonal trends (through different monthly intercepts). Since the mortality data for age and sex subgroups was available only on a yearly basis, the baseline was calculated as $D_{category,\;year}=\beta \cdot year+\alpha_{category}+\epsilon$ in those cases.

The variance of excess mortality was calculated by summing the variance due to the baseline uncertainty and variance due to Gaussian noise present even without the pandemic. See the Materials and Methods section in the article by Karlinsky and Kobak for more details [2]. Excess mortality prediction was deemed statistically significant if the z-score was larger than 2, where the z-score was calculated as $z=\frac{excess\;mortality}{\;\sqrt{Var(excess\;moratlity)}}$. All data modeling, data analysis and data visualization was performed using the R programming language (R language for statistical computing version 4.2.1, R Foundation for Statistical Computing, Vienna, Austria) [13].

## 3. Results

### 3.1. Excess Mortality on the National Level

#### 3.1.1. Excess Mortality for the Whole Population

On the country level, the total mortality in the 2020–2021 period was 119,706. Subtracting the modeled expected mortality from the recorded deaths, an excess mortality prediction of 14,963 was obtained. Translated into relative excess mortality and excess mortality death rate per 1000 people, the obtained values were 14.3% and 3.86, respectively.

#### 3.1.2. Excess Mortality with Respect to Age and Sex Differences

**Figure 1:**
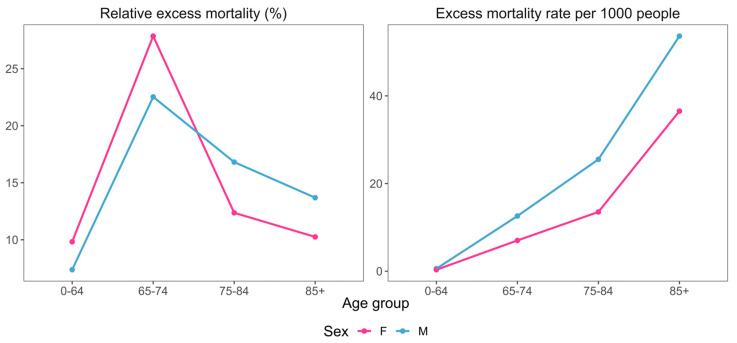
Country-level relative excess mortalities and excess mortality rates per 1000 people for different sex–age group combinations.

In addition to calculating excess mortality for the entire country population, we also estimated the excess across different age–sex subgroups. We observed an increased excess mortality rate in men (Figure 1, Table 1), with similar female/male excess mortality rates over different age groups.

In terms of age, the greatest relative increase in mortality appeared in the 65–74 years age group for both sexes. The excess mortality rates for both sexes indicate a continuous increase, with age resembling an exponential function (Figure 1, Table 1).

#### 3.1.3. Excess Mortality vs. Official COVID-19 Mortality

The ratio of expected mortality and official COVID-19 mortality can inform us on the quality of COVID-19 mortality tracking and the magnitude of indirect COVID-19 effects. The Croatian Institute of Public Health reports a total of 13035 deaths due to COVID-19 [14], resulting in an excess mortality/COVID-19 mortality ratio of 1.15. This points to an undercount of COVID-19 induced deaths.

### 3.2. Excess Mortality over Different Counties

#### 3.2.1. Excess Mortalities over Entire County Populations

**Figure 2:**
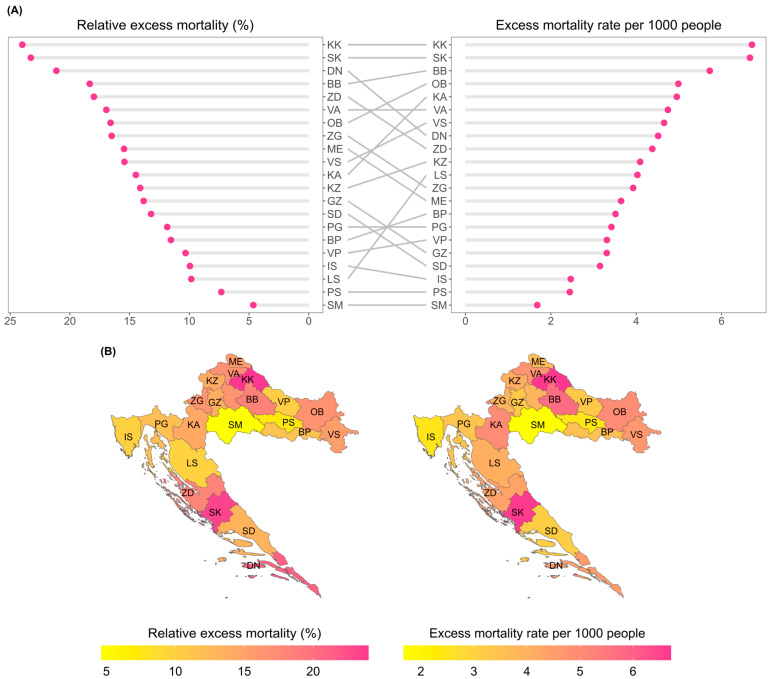
(**A**) County-level relative excess mortalities (left) and excess mortality rates per 1000 people (right) with lines in between connecting the positions representing the same counties for easier comparison of different metrics’ values. (**B**) Maps of relative excess mortalities and excess mortality rates across different counties.

This section analyzes the total excess mortality across Croatian counties. To reduce clutter on the charts, the county names are abbreviated, but the full names are available in Table A1. The results indicate large variability in the effect of the pandemic across counties (Figure 2). Both metrics unanimously show that the pandemic hit the KK and SK counties the most, and the effect was lowest for the PS and SM counties.

Figure 2 also shows that using only a single metric is often not enough to reach the correct conclusion on the impact of the pandemic. Using only the relative excess mortality or excess mortality rate would be enough if county rankings were always equal for both metrics (yielding horizontal connection lines in the middle). However, we can see that this is often not the case, and there are many counties for which the rankings differ strongly across the two metrics.

Counties for which the relative excess mortality is low and excess mortality rate is high (large positive slopes of gray lines in the middle, for example, the LS and KA counties) could indicate a high historical mortality baseline. Therefore, even a significant increase in excess mortality might not markedly change the relative excess mortality. A high historical mortality rate could be attributed to an older population demographic for such counties (Table 2). Conversely, counties with high relative excess mortality but low excess mortality rate suggest a low mortality baseline. Such examples are the GZ, SD and DN counties.

#### 3.2.2. Excess Mortality across Counties with Respect to Age and Sex Differences

**Figure 3:**
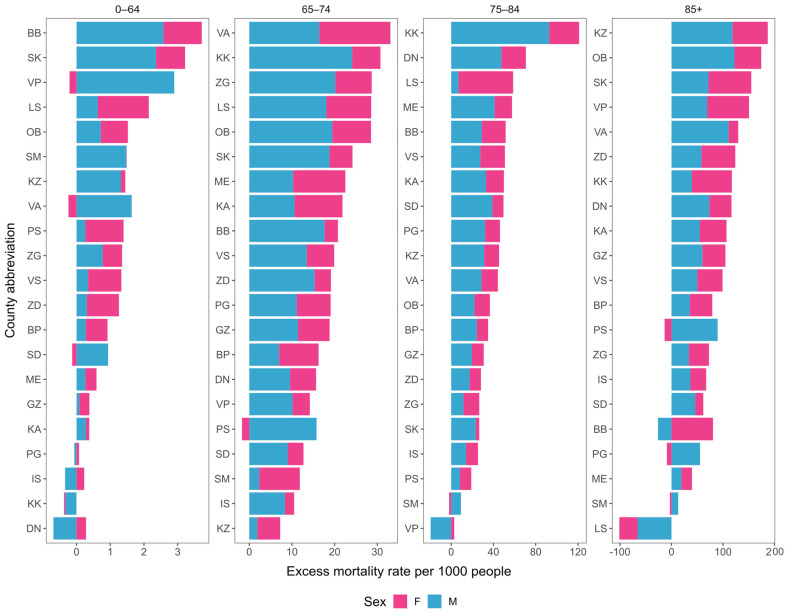
Comparison of specific excess mortality rates per 1000 people across counties and sexes for each of the age categories.

Just like with national-level data, we calculated excess mortality stratified by age and sex subgroups across different counties. In terms of excess mortality differences across sexes, the results again indicate a male bias in most counties, in agreement with the national trend (Figure 3). Stratifying according to age and sex also reveals that the main reason for the high excess mortality rate in the KK county is the large excess mortality rate in the 75–84 years age group, where it is almost twice as high as that of the county with the second highest value for that age group.

**Figure 4:**
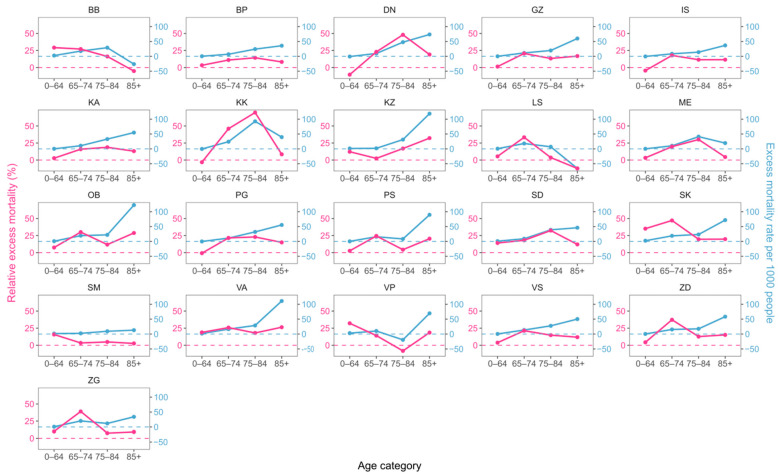
Comparison of two excess mortality metrics across counties for men in different age categories. Relative excess mortality is given in violet, and excess mortality rate per 1000 people in blue. Correspondingly colored dashed lines denote the zero value for each metric to facilitate comparison.

**Figure 5:**
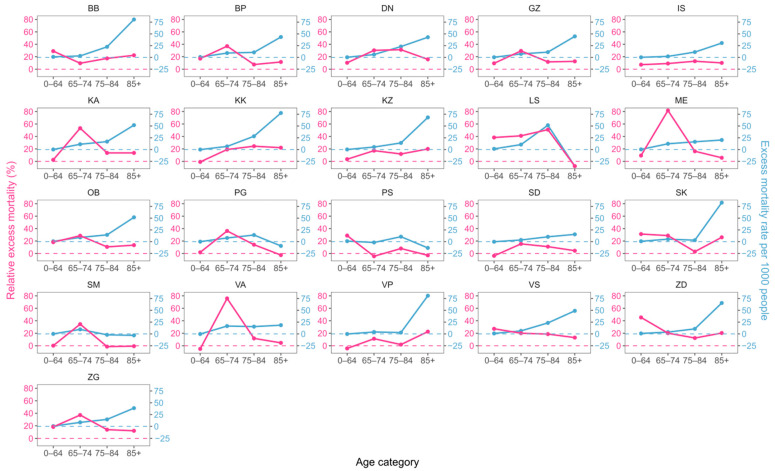
Comparison of two excess mortality metrics across counties for women in different age categories. Relative excess mortality is given in violet, and excess mortality rate per 1000 people in blue. Correspondingly colored dashed lines denote the zero value for each metric to facilitate comparison.

With respect to age, we again see an approximate exponential dependance of excess mortality rate on age for both sexes in most counties (Figure 4 and Figure 5). A notable exception to the general rule is the LS county, which displays negative excess percentages and mortality rates in the oldest age category.

Both Figure 4 and Figure 5 again point to a large difference in the behavior of the two excess mortality metrics, highlighting the necessity of simultaneously using both to avoid misconclusions.

## 4. Discussion

This study presents the excess mortality numbers for Croatia during the 2020–2021 COVID-19 pandemic. The excess was calculated both at the national and county levels, taking into account the age and sex structure of the population.

An excess mortality prediction of 14,963 was obtained for the whole country. Translated into cumulative relative excess mortality and excess mortality rate per 1000 people, that corresponds to values of 14.3% and 3.86, respectively. Other studies calculating excess mortality for Croatia over the same time span offer a wide range of estimates—from 12,205 to 22,900 [2,3,15,16,17,18]. These differences can be attributed to the usage of different statistical models and/or different time spans for the estimation of excess mortality. Also, all of those studies based their estimates on the Human Mortality Database [19,20] which (depending on the time of access and the time span in question) might include preliminary data subject to further revision. We obtained our mortality data from the *Croatian Health Statistics Yearbook* published by the Croatian Institute of Public Health [11]. Furthermore, their population estimates for Croatia were obtained from the United Nations World Population Prospects [4], and we used the 2021 Population Census data [12] for better population estimate accuracy.

In terms of comparison to other European countries, estimates of relative excess mortality based on the methodology by Karlinsky and Kobak show that Albania had the highest score of 34%, and Denmark and Iceland the lowest score of only 1% [17]. Croatia was positioned somewhere in the middle with a value of 16%, outperforming most Southeastern and Eastern European countries and scoring worse than most Western European countries. Since Western European countries have, on average, a higher per capita GDP than Eastern European countries, this indicates a possible role of a country’s economic status on the impact of COVID-19 [21,22].

A greater excess mortality rate was seen in men, in line with results from other studies for other countries [9,10] and Croatia [5]. The female/male excess mortality rates over different age groups on the national level were in agreement with another study on excess mortality across different countries [8]. An exponential relationship of excess mortality rate with age was observed, in accordance with the results from recent meta-analyses [6,7]. The relative increase in mortality was greatest for the 65–74 years age group, similar to the findings from another study focusing on excess mortality in Croatia, but only for the year 2020 [5].

Calculating the ratio of expected mortality and official COVID-19 mortality can inform us on the quality of COVID-19 mortality tracking and the magnitude of indirect COVID-19 effects. The obtained ratio of 1.15 is similar to estimates obtained by Karlinsky and Kobak using the same methodology, but accounting only for the year 2020 [2]. Such a value points to an undercount of COVID-19 induced deaths—either due to the imperfect counting of direct COVID-19 deaths, or due to a large increase in mortality from other causes induced by the COVID-19 pandemic [23,24].

Since the baseline for calculation of excess mortality includes the effect of mortality increase in winter due to influenza, the obtained estimate of excess mortality should be interpreted as excess mortality above the one observed in the presence of other seasonal infectious disease (such as influenza). Consequently, the excess mortality estimates presented here should be taken as the lower limit of excess mortality solely due to COVID-19. This is corroborated by recent studies indicating a substantial decrease of influenza levels during the beginning of the pandemic in the European region [25]. The recently published report by the Croatian Institute of Public Health on mortality causes in 2021 confirms the above statement, as there was only a single death due to influenza recorded during that entire year [14]. Nevertheless, we think that it is sensible to keep the effect of seasonal influenza in the baseline, as this approach gives us a number representing an additional mortality burden countries had to deal with on top of seasonal fluctuations.

The results on the county level underscore the necessity of simultaneously using both the relative excess mortality and excess mortality rate metrics when interpreting the impact of the pandemic. Using just one of them might be enough if county rankings were always equal for both metrics. However, this was often not the case, and there were many counties where the relative rankings differed strongly (Figure 2).

Counties for which the relative excess mortality was low and excess mortality rate was high could indicate a high historical mortality baseline in those counties. Conversely, a high excess percentage coupled with a low excess mortality rate could suggest a low county mortality baseline. All such counties have a younger demographic structure, probably because they contain large urban centers (cities of Zagreb, Split and Dubrovnik) which attract younger people due to a higher chance of finding work and better infrastructure for young families. Furthermore, such large cities have easier access to hospital care and better quality of hospitals, and both of these factors have been shown to decrease excess mortality rates [26,27,28].

Very low excess mortality values for the SM county deserve a special mention, since in December 2020, the county was hit by an earthquake of magnitude 6.3 on the Richter scale. The destruction of many homes, and/or fear of another disaster and aftershocks triggered migrations to other parts of the country [29,30]. The official number of people who left their homes is 2861, although the actual number is probably higher [31]. This could have contributed to low excess mortality in the region, since it decreased the population size, making the projected baseline overly high.

Just like with national-level data, we calculated excess mortality stratified by age and sex across different counties. In terms of excess mortality differences across sexes, the results indicate a male bias in most counties, in agreement with the national trend (Figure 3).

With respect to age, we again see an approximate exponential dependance of excess mortality on age for both sexes in most counties (Figure 4 and Figure 5). A notable exception to the general rule is the LS county, which displays negative excess percentages and mortality rates in the oldest age category.

A possible factor driving this deviation from expected behavior could be the effect of population density, as higher density means a greater probability of disease transmission. However, this parameter is not easily quantifiable, as it cannot be assessed by simply dividing the population count by the county area, since some counties may spread over large surfaces, in spite of most people living in fairly dense areas [32]. Consequently, “microscopic” effective densities have to be assessed, which, in addition to the local population density, also take into account additional factors such as the rate of daily migrations and percentage of people who use the public transportation system [32,33,34,35].

The LS County spans nearly 10% of Croatia’s surface, but contains only 1% of the total population, yielding a population density of just 7.99 people per square kilometer. Additionally, most people there live in houses, as opposed to large residential buildings containing many apartments. Consequently, in addition to low general density, the LS county also has a low effective (local) density, making it easier to impose social distancing. This could explain the unexpected low excess mortality rate in the oldest age group, in spite of this county having the highest proportion of people in that age group (largest number of people at risk) across all counties (Table 2).

Although excess mortality is a great metric for assessment of the overall magnitude of a crisis event, studies calculating excess mortality are faced with several methodological limitations. Estimating excess mortality requires defining a baseline level of expected mortality. The baseline value can be highly influenced by the choice of statistical model and/or the duration of the historical reference period, impacting the final excess mortality value [15]. The estimates are also influenced by how detailed the mortality data are (weekly, monthly or yearly mortality records). Although the total mortality counts across counties in our study were available on a monthly basis, the mortality data for different age and sex subgroups across counties were available only on a yearly basis, affecting the excess estimates. Finally, without detailed information on specific causes of death, excess mortality studies cannot capture the full complexity of how different factors contribute to mortality during a crisis. Further studies involving such information could elucidate the underlying causes for the difference between the recorded and estimated mortality during the COVID-19 pandemic.

## 5. Conclusions

Excess mortality emerged as a crucial indicator during the COVID-19 pandemic. Assuming the incidence of other diseases remained stable over the period of interest, it can give us an insight into the number of deaths directly or indirectly related to COVID-19. We calculated excess mortality on the national and subnational (county) levels across different age–sex subgroups. An excess mortality prediction of 14,963 was obtained for the whole country, in agreement with estimates from other studies ranging from 12,205 to 22,900.

The relationships of excess mortality with age and sex on the national level were in line with previous research, showing a greater mortality rate for men and an exponential increase in excess mortality rate with increasing age. The largest increase in mortality (largest relative excess mortality value) was observed in the 65–74 years age group.

On the county level, we showed that both relative excess mortality and excess mortality rate have to be used simultaneously in order to fully grasp the local effect of the pandemic. Different county rankings for each of these metrics were explained through differences in baseline mortality rates due to the distinct demographic structure of each county.

Although the age–sex trends across counties mostly followed the national trends, there were also exceptions to the general rule. These exceptions were discussed in terms of between-county differences in demographic structure, population density and special events that took place during the pandemic.

In summary, this study offers a comprehensive analysis of the effect of the COVID-19 pandemic on excess mortality in Croatia during the 2020–2021 period. Unlike previous studies involving Croatian data, it takes into account the age and sex differences on both the national and county level. Although the impact of the pandemic can be assessed using only absolute or relative measures of excess mortality, our results underline the necessity of using both metrics in order to be able to make correct and informed decisions.

## Figures and Tables

**Table 1:** Country-level excess mortalities for the entire population and different age–sex subgroups.

Age	Sex	Population	Excess Mortality	z-Score ^1^
0–64	M	1,503,611	878	1.98
0–64	F	1,498,983	525	2.15
65–74	M	230,234	2900	23.7
65–74	F	277,484	1949	7.27
75–84	M	103,737	2647	4.82
75–84	F	165,837	2243	2.23
85+	M	27,547	1477	5.36
85+	F	64,400	2352	2.28
All ages	Both sexes	3,871,833	14,963	5.09

^1^ Excess mortality prediction is deemed statistically significant if the z-score is larger than 2.

**Table 2:** Total county populations with percentages of population in each age category.

County	Population	0–64	65–74	75–84	85+
BB	101,879	76.8	13.9	6.9	2.4
BP	130,267	77.4	13.1	7.2	2.3
DN	115,564	77.5	13.0	6.9	2.6
GZ	767,131	79.3	11.6	6.8	2.3
IS	195,237	75.8	14.1	7.2	2.8
KA	112,195	75.5	13.9	7.6	3.1
KK	101,221	78.0	13.0	6.9	2.1
KZ	120,702	79.2	12.1	6.4	2.2
LS	42,748	73.7	13.9	8.7	3.6
ME	105,250	79.4	12.2	6.3	2.1
OB	258,026	78.0	13.3	6.7	2.0
PG	265,419	74.2	15.4	7.6	2.8
PS	64,084	77.1	13.3	7.3	2.2
SD	423,407	78.2	12.8	6.5	2.4
SK	96,381	72.6	15.5	8.5	3.3
SM	139,603	75.3	14.7	7.6	2.5
VA	159,487	79.2	12.1	6.6	2.1
VP	70,368	78.0	13.2	6.6	2.1
VS	143,113	77.2	13.6	7.1	2.2
ZD	159,766	76.0	14.0	7.5	2.5
ZG	299,985	78.7	12.9	6.4	2.0

## Data Availability

All data and code are available upon reasonable request from the corresponding authors.

## Appendix A

**Table A1:** Full names and abbreviations of Croatian counties.

County	Abbreviation
Bjelovar-Bilogora County	BB
Brod-Posavina County	BP
Dubrovnik-Neretva County	DN
Istria County	IS
Karlovac County	KA
Koprivnica-Križevci County	KK
Krapina-Zagorje County	KZ
Lika-Senj County	LS
Međimurje County	ME
Osijek-Baranja County	OB
Požega-Slavonia County	PS
Primorje-Gorski Kotar County	PG
Sisak-Moslavina County	SM
Split-Dalmatia County	SD
Varaždin County	VA
Virovitica-Podravina County	VP
Vukovar-Srijem County	VS
Zadar County	ZD
Zagreb	GZ
Zagreb County	ZG
Šibenik-Knin County	SK
